# Editorial: The molecular mechanisms of experience-dependent plasticity in invertebrates

**DOI:** 10.3389/fnbeh.2022.1123961

**Published:** 2023-01-05

**Authors:** Varvara E. Dyakonova, Etsuro Ito, Martin Giurfa

**Affiliations:** ^1^Koltzov Institute of Developmental Biology, Russian Academy of Sciences, Moscow, Russia; ^2^Department of Biology, Waseda University, Tokyo, Japan; ^3^Research Center on Animal Cognition, Center for Integrative Biology, Centre National de la Recherche Scientifique (CNRS), University of Toulouse, Toulouse, France; ^4^Institut Universitaire de France, Paris, France

**Keywords:** cognitive function, invertebrates, memory and learning, exercise, decision-making, food aversion, epigenetic mechanisms of memory, insulin

The goal of our Research Topic was to focus on novel discoveries concerning the molecular underpinnings of behavioral plasticity in invertebrates. Invertebrates are well-established models in the field of studies on the mechanisms of learning and memory. Essential contributions on the molecular bases of associative learning and memory formation have been made by animals as diverse as the sea hare *Aplysia californica* (Goelet et al., 1986; Kandel, 2001), the fruit fly *Drosophila melanogaster* (Heisenberg, 2003; Davis, 2005, 2011) and the honey bee *Apis mellifera* (Menzel, 1999; Giurfa, 2007) among others. The fact that these animals learn and memorize different types of information in a robust way that is amenable to standard laboratory protocols and that their nervous system is accessible to a wide variety of invasive approaches has expanded in a considerable way the research performed on the molecular mechanisms underlying their experience-dependent behavior. The advent of new techniques both for the analysis of behavior and for molecular analyses has brought a new dimension to these studies, which motivated the present Research Topic.

Five papers have been contributed to our Research Topic, dealing with front-rank scientific problems. Kemenes et al. reviewed the molecular mechanisms of associative plasticity in the pond snail *Lymnaea stagnalis*, highlighting the role of non-coding RNA and post-translational mechanisms upon single trial learning, Nakai et al. discussed the involvement of insulin molecular pathways in different forms of memory and food aversion in invertebrates, Lafon et al. focused on honey bees learning visual discriminations in a virtual-reality landscape and characterized learning-dependent Immediate Early Gene expression (IEG) in the bee brain, Dyakonova et al. reviewed the molecular basis and biological significance of evolutionary-conserved beneficial effects of exercise on behavior and brain plasticity and Yamagata et al. reported the discovery of a molecular mechanism based on the nutrient responding peptide hormone CCHamide-2 which mediates a direct interplay between brain reward and endocrine systems for long-term energy homeostasis.

Behavioral modulation of cognitive function is a subject of basic research with many crucial applications. Its importance comes from the need to understand the natural conditions affecting cognitive activity both in humans and other animals. Identifying the molecular mechanisms behind these effects may lead to the discovery of novel means of correction of cognitive and emotional status.

Various forms of behavioral modulation are considered in the papers published in our Research Topic. Some of these works (Nakai et al., Yamagata et al., Kemenes et al.,) consider cognitive-function modulation by hunger satiety status. For instance, Nakai et al. reviewed the role of insulin molecular cascades in up and down regulation of food aversive learning after short-term and prolonged starvation in nematodes, arthropods and mollusks. These findings on learned food aversion in invertebrates are significant also for understanding several human health problems, such as anorexia or recently emerged post-covid syndrome.

The single-trial learning paradigm described by Kemenes et al. for *L. stagnalis* is also based on a specific feeding state, i.e., a one-day long food deprivation. The review provides intriguing insights into involvement of non-coding RNA, mediating the activation of CREB and NO dependent cascades in memory consolidation (Kemenes et al.). What are the conditions for the formation of flashbulb memories? In the laboratory, pond snails are kept in rather impoverished environments. Would enrichment affect their readiness to form robust memories after a single trial conditioning? When information is redundant and animals regularly face many meaningless coincident stimuli under natural conditions, it is unlikely that any experienced coincidence will lead to a strong memory. This seems to be the case in honey bees, where consideration of environmental factors led to the conclusion that a single-trial odor conditioning may induce protein-synthesis dependent long-term memories. If experiments are performed in the absence of predators (hornets) and using true nectar foragers, long-term memories are formed after a single trial instead of three (Villar et al., 2020).

In *D. melanogaster* (Yamagata et al.), a direct link between the satiety status and the activity of dopaminergic neurons mediating reward signals necessary for appetitive learning and long-term memory (LTM) is discovered. The protostome peptide CCHamide-2 (CCHa2) was found to convey nutrient signals and to be the key molecule responsible for this link (Yamagata et al.).

The review of Dyakonova et al., deals with another form of behavioral modulation of cognitive status in various invertebrates, which is mediated by previous intense locomotion. The authors consider the possible adaptive significance of behavioral and cognitive effects of exercise, which is also well known in vertebrates, including humans. The authors suggest that a general homeostatic switch from stability to higher plasticity, seen at the behavioral, metabolic and genetic levels, and epigenetically transferred to the first generation (Mezheritskiy and Dyakonova, 2022), may serve adaptation to novel environments.

Finally, the research paper of Lafon et al., investigating visual learning by bees in a virtual-reality setup provides a clear illustration of the powerful impact of behavioral context – and more specifically of learning type - on molecular events underlying associative learning. The article also points to gaps in our understanding of the links between neuronal activity, IEGs expression and plasticity of behavior. The authors demonstrate unusual down-regulation of ERG-1 during visual discriminating learning in the bee brain. This finding contrasts with the more common phenomenon of IEGs up-regulation upon enhanced neuronal activity during cognitive activation (Geng et al., 2022). Moreover, results of the same group obtained in a similar learning experiment but under different conditions of movement control provided to the animals (2d 3d handling of virtual stimuli in virtual reality), showed a different pattern of IEG expression after visual learning. The authors suggest several possible explanations for this phenomenon. In addition, it is also known that IEGs expression can be induced by double-strand DNA breaks in neurons and oxidative stress (Madabhushi et al., 2015). Hence events affecting neuronal oxidant/antioxidant systems may impact IEGs expression in addition to neuronal activity, enhancing or preventing the IEG expression caused by DNA breaks. In that sense, model protostomes seem to be ideal objects to investigate the complex interplay between behavioral and molecular plasticity and their costs in terms of DNA damage.

Protostomes have been used for decades as research objects in neuroethology. The set of novel molecular tools available and the deeper knowledge gained on their biology thanks to the sequencing of several invertebrate genomes has opened new research avenues that are well illustrated by the present collection of articles.

## Author contributions

All authors listed have made a substantial, direct, and intellectual contribution to the work and approved it for publication.
